# The Journey to Hepatitis C Elimination in Taiwan: Insights From the Hemodialysis Population

**DOI:** 10.1093/ofid/ofag039

**Published:** 2026-01-29

**Authors:** Ming-Yan Jiang, Yi-Chan Lee, Tsung-Hsueh Lu

**Affiliations:** Department of Public Health, National Cheng Kung University, Tainan, Taiwan; Renal Division, Department of Internal Medicine, Chi Mei Medical Center, Tainan, Taiwan; Department of Pharmacy, Chia Nan University of Pharmacy and Science, Tainan, Taiwan; Institute of Health Policy and Management, College of Public Health, National Taiwan University, Taipei, Taiwan; Department of Public Health, National Cheng Kung University, Tainan, Taiwan

**Keywords:** direct-acting antivirals (DAA), end-stage kidney disease, hemodialysis, hepatitis c virus (HCV), microelimination

## Abstract

**Background:**

Hemodialysis patients are a priority population for hepatitis C virus (HCV) elimination in Taiwan, where HCV prevalence and end-stage kidney disease burden are high. The introduction of direct-acting antivirals (DAAs) has accelerated elimination efforts. This study evaluated HCV care cascade and identified gaps among Taiwan's hemodialysis patients in the DAA era.

**Methods:**

We conducted a retrospective cohort study using National Health Insurance Research Database from 2015 to 2021. Adult patients receiving long-term hemodialysis were included. HCV infection was defined as positive RNA test, and treatment initiation as receipt of ≥7 days of DAA therapy. We assessed the care cascade, annual and cumulative treatment rates, and disparities by demographic and facility characteristics.

**Results:**

Among 14 755 HCV antibody-positive patients, 48.2% underwent confirmatory RNA testing. Of 4783 viremic patients, 73.4% initiated DAA therapy. The greatest attrition occurred at the RNA confirmation step. Testing rates were lower among males (47% vs 50% in females), older adults, patients treated in tertiary hospitals (46%), and those in metropolitan areas (48% vs 52% rural). Regional variation was substantial, ranging from 38% to 41% in Eastern, Central, and Taipei regions to 67% in Southern Taiwan. Annual treatment initiation rose from 3.0% in 2017% to 50% in 2019, and then declined to 33% in 2021. Cumulative treatment increased from 10% before 2018% to 73.4% by 2021.

**Conclusions:**

HCV care among hemodialysis patients in Taiwan has improved substantially in the DAA era. The major barrier is incomplete RNA testing. Addressing this gap is essential to sustain progress and achieve HCV elimination in this high-risk population.

Hepatitis C virus (HCV) infection remains a major global health challenge [1, 2], including in Taiwan [3, 4]. In 2016, the World Health Organization (WHO) set a goal to eliminate HCV as a public health threat by 2030 [5]. In response, Taiwan launched a comprehensive national strategy aiming to eliminate HCV by 2025, 5 years ahead of the WHO target [6–8]. This strategy focused on early detection, expanded access to direct-acting antivirals (DAAs), and optimized care delivery across diverse populations [6–8].

A pivotal advancement in Taiwan's national strategy was the phased introduction and expansion of DAA therapy coverage through the National Health Insurance (NHI) system [6–8]. The NHI covered the full cost of HCV diagnosis including anti-HCV antibody testing and RNA confirmation, and the DAA medications, effectively eliminating patient financial barriers such as copayments or coinsurance, a model vital for achieving high treatment uptake. To further enhance treatment access and equity, Taiwan systematically decentralized HCV care by integrating screening and treatment into local health facilities and empowering nonspecialist healthcare providers, including general practitioners, to prescribe DAAs. Specific efforts were made to encourage provider participation through educational initiatives and to optimize service delivery via microelimination programs targeting high-risk groups such as hemodialysis patients. While DAA therapy in Taiwan was initially prescribed predominantly by gastroenterology/hepatology specialists, an increasing proportion of prescriptions were later provided by trained nonspecialist physicians as decentralized treatment pathways and educational initiatives expanded. These system-level changes, supported by robust national surveillance for data-driven policy adjustments, provided the foundation for effective care delivery [6–8].

Given Taiwan's high burden of treated end-stage kidney disease, which has the highest global prevalence at 3806 cases per million population in 2022 [9], and the high prevalence of HCV in this population [10–12], hemodialysis patients have been identified as a priority for elimination efforts [6–8]. Local initiatives have demonstrated the success of targeted approaches [13, 14]. For example, the Changhua Integrated Program to Stop HCV Infection (CHIPS-C) program, a collaborative care model led by the local health authority, showed that targeted microelimination strategies increased DAA treatment uptake among dialysis patients in Changhua County from 25% to nearly 90% [13]. This program utilized a cross-sectoral, multidisciplinary partnership to integrate HCV care into existing health services, simplify care processes, and optimize delivery to affected populations [13]. Similarly, the ERASE-C Campaign in Southern Taiwan, which combined mass screening with on-site group treatment across 18 hemodialysis units, demonstrated the feasibility of achieving HCV microelimination in dialysis facilities [14].

Prior to the DAA era, the treatment rate for HCV among hemodialysis patients in Taiwan was historically low, with only 6% receiving interferon-based therapy [15]. Despite the recent progress demonstrated by local microelimination programs [13, 14], uncertainties remain regarding the national implementation of HCV therapy among the dialysis population. It is unclear whether expanded policies have translated into equitable access and improved treatment uptake nationwide. Using the National Health Insurance Research Database (NHIRD), this study aimed to characterize trends in the HCV care cascade among hemodialysis patients in Taiwan and to identify gaps in diagnostic testing and treatment uptake from the beginning of the DAA era in 2015 through 2021.

## METHODS

### Data Source and Study Population

We conducted a retrospective cohort study using the Taiwan NHIRD, a nationwide claims database maintained by the Ministry of Health and Welfare [16, 17]. The NHI operates a single-payer, universal coverage system, covering over 99% of Taiwan's population, including all citizens and legal foreign residents. The NHIRD is derived from this system, containing deidentified demographic information, longitudinal medical records, prescription claims, and selected laboratory test results from the National Health Insurance Laboratory Database [18].

We used the viral hepatitis dataset, which identifies patients with hepatitis B or C using ICD-9-CM (070, 570–573) and ICD-10-CM (B15–B19, K70–K77) codes between 2015 and 2021 (Supplementary Figure 1). Eligible participants were adults (≥18 years) who had received hemodialysis for at least 3 consecutive months, identified by a specific reimbursement code (*n* = 46 644). We excluded patients who died in 2015–2016 (*n* = 4201) and those who received HCV treatment prior to dialysis initiation (*n* = 585). We further excluded 3356 patients who lacked both anti-HCV antibody and HCV RNA test results, resulting in a final analytic cohort of 38 502 hemodialysis patients (Supplementary Figure 1).

## Supplementary Material

ofag039_Supplementary_Data

## Figures and Tables

**Figure 1.**
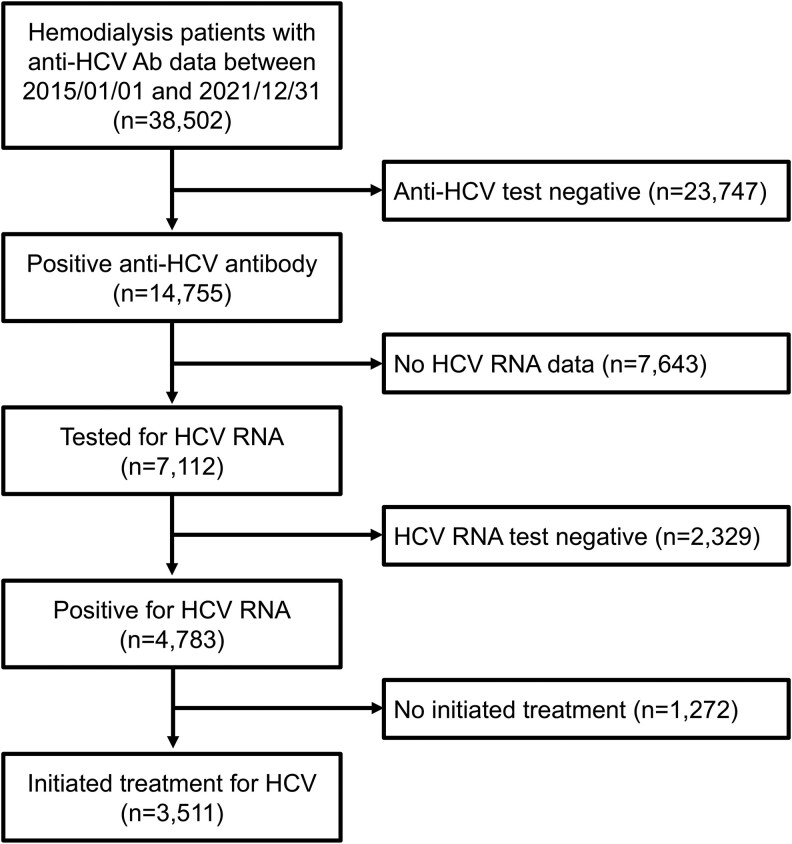
Hepatitis C virus (HCV) care cascade among hemodialysis patients in the study population.

**Table 1.** Care Cascade for Hepatitis C Virus (HCV) Infection Among Hemodialysis Patients

	Confirmatory Test				Treatment Initiation			
	HCV Ab Positive (*N* = *a*)	HCV RNA Tested (*N* = *b*)	% (*b*/*a*)	Crude OR (95% CI)	HCV RNA-Positive (*N* = *c*)	Initiated Treatment (*N* = *d*)	% (*d*/*c*)	Crude OR (95% CI)
Characteristic								
Total	14 755	7112	48.2		4783	3511	73.4	
Sex								
Male	7888	3673	46.6	0.88 (.82–.94)***	2502	1841	73.6	1.02 (.90–1.16)
Female	6867	3439	50.1	1.00 (ref)	2281	1670	73.2	1.00 (ref)
Age (years)	63.7 ± 12.3	62.7 ± 11.6	–	0.99 (.98–.99)***	62.7 ± 11.4	61.7 ± 11.1	–	0.97 (.97–.98)***
Age group								
<45	998	463	46.4	0.79 (.69–.90)***	288	224	77.8	0.96 (.72–1.29)
45–64	6745	3535	52.4	1.00 (ref)	2429	1906	78.5	1.00 (ref)
≥65	7012	3114	44.4	0.73 (.68–.78)***	2066	1381	66.8	0.55 (.48–.63)***
Facility type								
Tertiary hospital	1313	603	45.9	1.00 (ref)	333	242	72.7	1.00 (ref)
Secondary hospital	3564	1739	48.8	1.23 (1.08–1.40)**	1176	879	74.7	1.11 (.85–1.47)
Primary hospital	3274	1639	50.1	1.20 (1.06–1.36)**	1116	795	71.2	0.93 (.71–1.22)
Freestanding unit	6604	3131	47.4	1.20 (1.07–1.35)**	2158	1595	73.9	1.07 (.82–1.38)
NHI region^a^								
Taipei	3417	1396	40.8	1.00 (ref)	935	645	69	1.00 (ref)
Northern	1988	947	47.6	1.40 (1.25–1.56)***	692	543	78.4	1.64 (1.30–2.06)***
Central	3181	1301	40.9	0.97 (.88–1.06)	787	610	77.5	1.55 (1.25–1.93)***
Southern	2578	1734	67.3	3.32 (2.96–3.71)***	1207	891	73.8	1.27 (1.05–1.53)*
Kaopin	2839	1445	50.9	1.33 (1.20–1.47)***	969	681	70.3	1.06 (.87–1.29)
Eastern	752	289	38.4	0.73 (.62–.86)***	193	141	73.1	1.22 (.86–1.73)
Urbanization								
Rural township	1875	966	51.5	1.34 (1.19–1.50)***	626	487	77.8	1.61 (1.29–2.02)***
Suburban township	3298	1656	50.2	1.15 (1.05–1.27)**	1137	854	75.1	1.39 (1.16–1.67)***
Urban township	5685	2619	46.0	1.02 (.94–1.10)	1805	1338	74.1	1.32 (1.12–1.55)***
Metropolitan	3897	1871	48.0	1.00 (ref)	1215	832	68.4	1.00 (ref)

Abbreviations: Ab, antibody; OR, odds ratio; CI, confidence interval; NHI, National Health Insurance.

^a^NHI care region classification was based on administrative divisions.

*P < .05; **P < .01; **P < .001.

**Figure 2.**
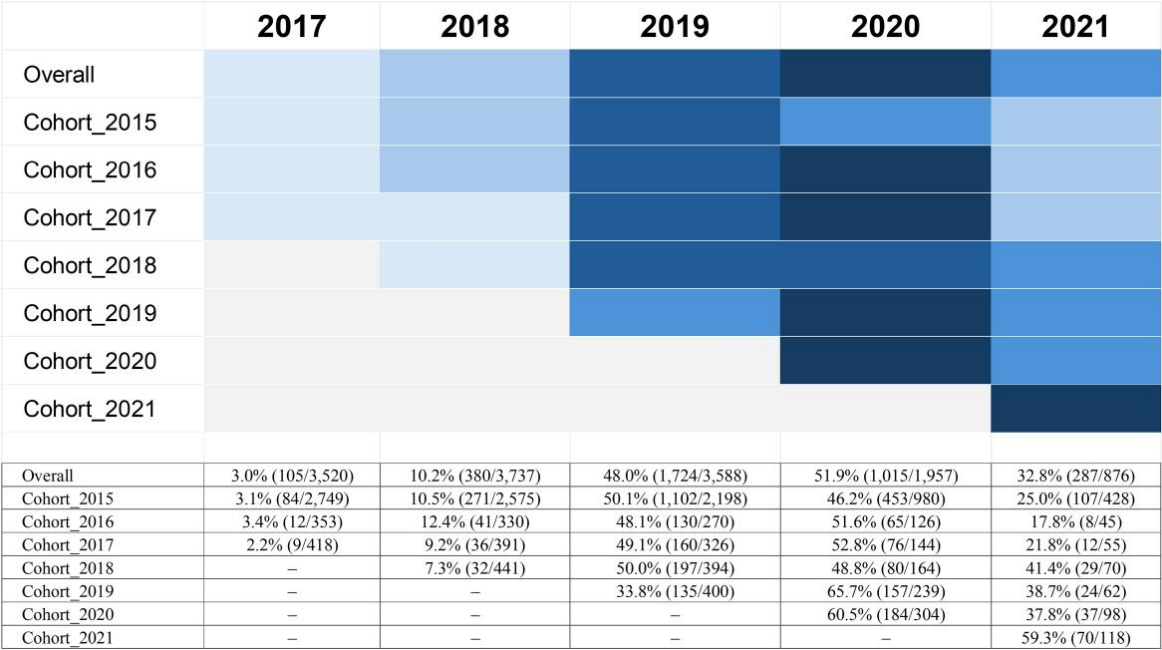
Annual Hepatitis C virus (HCV) treatment initiation rates stratified by cohort year (2015–2021) and treatment year (2017–2021). This heatmap visualizes the annual proportion of untreated HCV viremic patients in the long-term hemodialysis cohort who initiated direct-acting antiviral (DAA) therapy in a given calendar year. The numerator (*N*) is the number of patients initiating DAA treatment in that year. The denominator (*N*) is the total number of untreated patients with HCV at the start of that year. The statistical significance of the linear trend in annual treatment initiation rates across all cohorts was confirmed using the Cochran–Armitage trend test (*P* < .001).

**Table 2.** Comparison of Annual HCV Treatment Initiation Rates Between 2018 and 2019, Stratified by Patient and Facility Characteristics

	2018 N/N (%)	2019 N/N (%)	RR (95% CI)
Characteristic			
Total	380/3737 (10.2)	1724/3588 (48.0)	4.7 (4.2–5.3)
Sex			
Male	228/1949 (11.7)	888/1840 (48.3)	4.1 (3.6–4.8)
Female	152/1788 (8.5)	836/1748 (47.8)	5.6 (4.7–6.7)
Age group (years)			
<45	13/253 (5.1)	114/245 (46.5)	9.1 (5.1–16.1)
45–64	218/2058 (10.6)	1002/1923 (52.1)	4.9 (4.2–5.7)
≥65	149/1426 (10.4)	608/1420 (42.8)	4.1 (3.4–4.9)
Facility type			
Tertiary hospital	31/273 (11.4)	143/254 (56.3)	5.0 (3.4–7.3)
Secondary hospital	108/945 (11.4)	428/870 (49.2)	4.3 (3.5–5.3)
Primary hospital	75/824 (9.1)	407/844 (48.2)	5.3 (4.1–6.8)
Freestanding unit	166/1695 (9.8)	746/1620 (46.0)	4.7 (4.0–5.6)
NHI Region			
Taipei	55/743 (7.4)	288/721 (39.9)	5.4 (4.0–7.2)
Northern	36/564 (6.4)	305/573 (53.2)	8.3 (5.9–11.8)
Central	80/611 (13.1)	345/576 (59.9)	4.6 (3.6–5.8)
Southern	134/935 (14.3)	421/851 (49.5)	3.5 (2.8–4.2)
Kaopin	61/736 (8.3)	317/719 (44.1)	5.3 (4.0–7.0)
Eastern	14/148 (9.5)	48/148 (32.4)	3.4 (1.9–6.2)
Urbanization			
Rural township	45/462 (9.7)	253/470 (53.8)	5.5 (4.0–7.6)
Suburban township	89/864 (10.3)	410/850 (48.2)	4.7 (3.7–5.9)
Urban township	157/1428 (11.0)	642/1341 (47.9)	4.4 (3.7–5.2)
Metropolitan	89/983 (9.1)	419/927 (45.2)	5.0 (4.0–6.3)

Abbreviations: HCV, Hepatitis C virus; RR, rate ratio; CI, confidence interval; *n*, number of patients initiating treatment; *N*, number of untreated patients with HCV at the start of each year; NHI, National Health Insurance.

**Table 3.** Cumulative Treatment Initiation Rate Stratified by Observational Cohorts

Cohort	HCV RNA-Positive Patients (a)	2017 *N* (%)	2018 *N* (%)	2019 *N* (%)	2020 *N* (%)	2021 *N* (%)
Overall	4783	105 (2.2)	485 (10.1)	2209 (46.2)	3224 (67.4)	3511 (73.4)
2015	2749	84 (3.1)	355 (12.9)	1457 (53.0)	1910 (69.5)	2017 (73.4)
2016	353	12 (3.4)	53 (15.0)	183 (51.8)	248 (70.3)	256 (72.5)
2017	418	9 (2.2)	45 (10.8)	205 (49.0)	281 (67.2)	293 (70.1)
2018	441	–	32 (7.3)	229 (51.9)	309 (70.1)	338 (76.6)
2019	400	–	–	135 (33.8)	292 (73.0)	316 (79.0)
2020	304	–	–	–	184 (60.5)	221 (72.7)
2021	118	–	–	–	–	70 (59.3)

HCV, hepatitis C virus.

*N* = number of patients initiating treatment by year.

The denominator for each cumulative rate (%) is the total number of HCV RNA-positive patients in each cohort (a).

## Data Availability

Because uploading laboratory data for the National Health Insurance Laboratory Database is encouraged but not mandatory [18], its completeness varies systematically across health care settings in our study population. Specifically, while overall availability of anti-HCV antibody results was 92%, the completeness ranged from 87.0% in freestanding clinics to 99.0% in tertiary hospitals, with primary and secondary hospitals at 95.0% and 96.6%, respectively (Supplementary Table 1). HCV RNA data availability among those tested remained consistent across facilities, ∼85% (Supplementary Table 1).

## Definitions of Variables

Anti-HCV antibody and HCV RNA test results were obtained from the Laboratory Database [18]. Results recorded as text were categorized as positive (“positive” or “+”) or negative (“negative,” “–,” “borderline,” or “gray zone”). Numeric results were classified as positive based on test-specific thresholds (eg, ≥1 S/CO, ≥1 COI, ≥2 S/N for antibody; ≥10–15 IU/mL for RNA). When unit or reference range information was missing, predefined cutoffs (≥2 for antibody, ≥15 IU/mL for RNA) were applied. Patients with RNA results but missing antibody data were assumed anti-HCV antibody positive. Detailed algorithms are provided in Supplementary Figures 2 and 3. HCV treatment initiation was defined as receipt of DAA therapy for ≥7 days, identified through NHI order codes for DAA medications (Supplementary Table 2). This 7-day threshold was selected because claims in the NHIRD are typically issued in 7-day units, and a 7-day fill indicates a clear intent to initiate a full course of therapy while minimizing the inclusion of erroneously issued claims.

## Covariates

Patients were stratified by sex, age (<45, 45–64, ≥65 years), and year of entry into long-term hemodialysis. The 2015 cohort included both incident and prevalent patients; subsequent yearly cohorts included only incident cases. Patients were followed until 31 December 2021, to ascertain HCV treatment status. Dialysis facilities were categorized by hospital level (tertiary, secondary, primary, and freestanding clinics) and by geographic region, based on the 6 NHI divisions (Taipei, Northern, Central, Southern, Kaoping, and Eastern). Facility urbanization was classified as metropolitan, urban, suburban, or rural townships, using official government definitions.

## Statistical Analysis

Descriptive statistics were used to summarize patient characteristics and HCV care cascade outcomes. Continuous variables are presented as mean ± standard deviation, and categorical variables as counts and percentages.

For the care cascade, we calculated the proportion of patients at each step (anti-HCV antibody positivity, RNA confirmation, and DAA initiation among viremic patients). These cascade proportions were calculated using a serial denominator, where each step's denominator consists of only those patients who completed the prior step. Differences in RNA testing and treatment initiation across demographic and facility characteristics were evaluated using logistic regression, with results reported as odds ratios (ORs) and 95% confidence intervals (CIs).

Temporal trends in treatment initiation were assessed by calculating annual and cumulative initiation rates. The annual treatment initiation rate was defined as the number of patients who initiated therapy in a given year divided by the number of untreated viremic patients at the beginning of that year. The cumulative rate represented the overall proportion of viremic patients who initiated therapy during the study period. The statistical significance of the linear trend over time was formally tested using the Cochran–Armitage trend test. Furthermore, rate ratios (RRs) with 95% CIs were estimated by comparing the initiation rate in each year with the rate of the preceding year to assess the magnitude of year-on-year change.

Subgroup analyses were conducted to explore disparities in RNA testing and treatment initiation by sex, age group, dialysis facility type, urbanization level, and geographic region.

All statistical analyses were performed using SAS version 9.4 (SAS Institute, Cary, NC). A two-tailed *P* < .05 was considered statistically significant.

## RESULTS

### Cascade of Care in the Overall Study Population

Between 2015 and 2021, 41,858 adults received chronic hemodialysis; of these, 38 502 (92.0%) had anti-HCV antibody results available and were included in the final cohort (Supplementary Table 1). Anti-HCV antibody data availability was high overall, showing comparable completeness across sex and age groups. However, completeness varied systematically by facility type (detailed in Supplementary Table 1). Among 8315 patients with RNA testing ordered, valid results were available for 7112 (85.5%), with comparable completeness across subgroups (Supplementary Table 1).

Overall, 14 755 patients (38.3%) were HCV antibody-positive (Figure 1). Of these, 7112 (48.2%) underwent confirmatory HCV RNA testing, 4783 (67.3%) were viremic, and 3511 (73.4% of viremic patients) initiated DAA therapy (Figure 1).

### Care Cascade by Sex and Age Group

Males had slightly lower RNA testing uptake than females (47% vs 50%; OR .88, 95% CI .82–.94), but treatment initiation among viremic patients was similar (73% vs 74%; OR 1.02, 95% CI .90–1.16) (Table 1).

Testing rates also varied across age groups: 46% in patients <45 years, 52% in those 45–64 years, and 44% in those ≥65 years. Compared with middle-aged adults, younger (OR .79, 95% CI .69–.90) and older adults (OR 0.73, 95% CI .68–.78) were less likely to undergo RNA testing. Treatment initiation was higher in younger and middle-aged adults (both 78%) but lowest in older adults (67%). Compared with middle-aged adults, older adults were significantly less likely to start therapy (OR 0.55, 95% CI .48–.63) (Table 1).

### Care Cascade by Dialysis Facility and Location

RNA testing uptake varied by facility type, region, and degree of urbanization (Table 1). Testing was lowest in tertiary hospitals (46%) and highest in primary hospitals (50%). Compared with tertiary hospitals, patients treated in secondary hospitals, primary hospitals, and freestanding clinics all had higher odds of undergoing RNA testing (Table 1).

Marked regional differences were also observed: uptake was 38%–41% in Eastern, Central, and Taipei regions but reached 67% in Southern Taiwan (Table 1). Compared with Taipei, the odds of testing were significantly higher in Northern (OR 1.40, 95% CI 1.25–1.56), Southern (OR 3.32, 95% CI 2.96–3.71), and Kaoping (OR 1.33, 95% CI 1.20–1.47) regions. Testing was lower in metropolitan areas (48%) than in suburban (50%) or rural townships (52%) (Table 1).

Among viremic patients, treatment initiation rates were similar across facility types (71%–75%) (Table 1). However, regional disparities persisted: initiation was lowest in Taipei (69%) and highest in Northern and Central regions (78%). Compared with Taipei, the odds of treatment were higher in Northern (OR 1.64, 95% CI 1.30–2.06), Central (OR 1.55, 95% CI 1.25–1.93), and Southern (OR 1.27, 95% CI 1.05–1.53) regions. Patients in metropolitan areas were less likely to initiate therapy (68%) than those in urban (74%), suburban (75%), and rural settings (78%) (Table 1).

### Annual Treatment Initiation by Cohort

Annual treatment initiation was minimal in 2017 (3.0%) and 2018 (10.2%), rose sharply in 2019 (48.0%), and peaked in 2020, before declining in 2021, when approximately one-third of viremic patients initiated treatment (*P* for trend < .001) (Figure 2).

Across cohorts, patients who started dialysis between 2015 and 2018 showed parallel increases, with initiation rates reaching nearly 50% in 2019. The 2019 cohort achieved 33% initiation in the first year and two-thirds by 2020, but this declined to 38.7% in 2021. The 2020 cohort followed a similar trajectory, while the 2021 cohort had the highest first-year initiation rate (nearly 60%) (Figure 2).

### Comparison of Annual Treatment Initiation Between 2018 and 2019

The rate of treatment initiation in 2019 was 4.7-fold higher than in 2018 (RR 4.7, 95% CI 4.2–5.3) (Table 2). The increase was greater in females (RR 5.6, 95% CI 4.7–6.7) than in males (RR 4.1, 95% CI 3.6–4.8), and most pronounced in patients <45 years (RR 9.1, 95% CI 5.1–16.1). Similar increases were observed across facility types (RR range 4.3–5.3). The Northern region showed the largest regional increase (RR 8.3, 95% CI 5.9–11.8). Trends were consistent across urbanization levels (RR 4.4–5.5) (Table 2).

### Trends in the Care Cascade

From 2017 to 2021, cumulative uptake of confirmatory RNA testing increased from 15% to 48%, while treatment initiation among viremic patients rose from 2% to 73% (Table 3; Supplementary Figure 4, Supplementary Tables 3–4). Among the 4783 viremic patients, 2749 (57.5%) originated from the 2015 cohort, indicating that many remained untreated before DAAs became widely available. Cumulative treatment initiation was only 10% before 2018, but scaled up to nearly 50% in 2019. By the end of the study period in 2021, the cumulative treatment initiation rate among viremic hemodialysis patients had reached a substantial 73%, demonstrating a significant and positive national trend (Table 3; Supplementary Figure 4, Supplementary Tables 3–4).

Across cohorts, treatment initiation reached ∼70% by 2021, with the 2019 and 2020 cohorts achieving comparable uptake within 2 years of dialysis initiation (Table 3).

## DISCUSSION

In this large, nationally representative cohort of more than 38 000 hemodialysis patients in Taiwan, we observed substantial progress in the HCV care cascade between 2015 and 2021. The sharp rise in treatment uptake, particularly the 4-fold increase from 2018 to 2019, underscores the transformative impact of Taiwan's holistic national strategy, including expanded treatment access, decentralized care, and targeted microelimination efforts, on HCV care in this population. By 2021, over 70% of viremic patients had received treatment. While this figure has not yet reached the WHO's 2030 target of ≥80% treatment coverage among viremic patients, this rapid scale-up demonstrates that Taiwan is on track for microelimination in the hemodialysis population.

Despite these achievements, important gaps remain. The greatest loss in the cascade occurred at the HCV RNA confirmation stage, where fewer than half of seropositive patients were tested. This finding aligns with barriers reported globally, where the need for a 2-step testing approach is recognized as a major source of loss-to-follow-up [19–24]. Because confirmatory testing is the essential gateway to treatment, this step represents the dominant clinical barrier to cure. Although Taiwan mandates baseline and annual anti-HCV screening for hemodialysis patients, confirmatory RNA testing has historically not been routine, delaying definitive diagnosis and timely treatment initiation. Bridging this gap will require streamlined diagnostic pathways, such as reflex RNA testing automatically triggered by a positive antibody result, standing orders integrated into dialysis workflows, and point-of-care (POC) RNA testing to minimize delays and reduce additional patient visits, as demonstrated in successful microelimination models in Australia and other settings [19, 20, 25–28].

Our findings reveal notable disparities in the HCV care cascade by facility type, geographic region, and urbanization level. Surprisingly, HCV RNA testing rates were lowest among patients treated at tertiary hospitals, despite these facilities having greater access to specialist care. This may reflect operational challenges and competing clinical priorities in high-volume, specialized settings, where the inherent complexity of treating diverse patient needs often results in the deprioritization of routine public health tasks like HCV screening and linkage to care [29, 30]. In contrast, primary hospitals achieved the highest testing rates, likely due to simpler care pathways and closer integration of HCV management into routine dialysis workflows. Similar patterns were observed in treatment initiation, with patients in metropolitan areas and tertiary facilities less likely to start therapy compared to those in rural or primary hospital settings. These findings support the growing global evidence that decentralized and simplified care models lead to significantly higher treatment uptake compared to traditional hospital-based care [31]. These parallel trends suggest that barriers such as fragmented care coordination, workforce limitations, and high patient turnover may hinder both diagnostic testing and treatment uptake. Targeted interventions, such as standardized HCV care pathways and enhanced linkage-to-care initiatives, are needed to address these disparities and ensure equitable access across all regions and facility types [32–34].

Our findings are consistent with reports from Taiwan's local microelimination efforts in dialysis units and extend these observations to the national scale [13, 14]. By quantifying where attrition occurs and identifying subgroups and facility types with persistent gaps, this study provides a comprehensive view of the national HCV care cascade. The annual treatment initiation rate peaked in 2019–2020 and then declined in 2021. The initial surge closely followed the nationwide expansion of DAA reimbursement, demonstrating how universal access can drive immediate gains in treatment coverage [35, 36]. Importantly, treatment initiation among newly identified patients in 2021 remained high, with nearly 60% starting therapy in their first year, indicating maintained treatment capacity for incident cases. While the 2021 decline coincided with the COVID-19 pandemic [37–40], our data suggest that this reduction was not entirely pandemic-driven. Rather, the trajectory likely reflects the natural progression of treatment uptake. In the early years of DAA reimbursement expansion, patients who were already diagnosed and awaiting treatment were rapidly initiated on therapy. As this backlog cleared, the remaining untreated population likely consisted of individuals facing additional barriers, such as advanced comorbidities, contraindications, or reluctance to undergo treatment [41]. Moving forward, the focus of elimination efforts must shift from broad access to targeted strategies that engage these harder-to-reach patients through sustained provider outreach, patient education, and individualized care plans addressing medical, psychosocial, and logistical challenges.

The success achieved in the hemodialysis population, a highly engaged yet high-risk group, offers valuable lessons for Taiwan's broader HCV elimination efforts. Microelimination programs such as CHIPS-C and ERASE-C demonstrates the effectiveness of decentralized or colocated test-and-treat models within dialysis settings [13, 14]. These models integrate screening, confirmatory testing, and treatment directly into the routine dialysis workflow, thereby simplifying the care pathway and minimizing patient loss to follow-up. Although most DAA treatments were still initiated by specialists during the early DAA era, the expansion of provider training and streamlined care pathways enabled a growing proportion of prescriptions to be issued by nonspecialist clinicians, further facilitating integrated care within dialysis units. Key lessons for national elimination strategies include: (1) the importance of colocated and decentralized care pathways to ensure equitable access; (2) the critical role of removing financial barriers to DAA therapy; and (3) the substantial impact of streamlined diagnostic protocols in addressing the RNA testing bottleneck. These decentralized principles are highly adaptable and should be prioritized for other high-risk, lower-engagement populations, such as people who inject drugs or incarcerated individuals. Ongoing efforts must focus on translating the system-level successes demonstrated in the dialysis setting to community-based and decentralized models.

Strengths of this study include its large national cohort, longitudinal follow-up, and linkage of laboratory and prescription data, which allowed for a robust assessment of cascade gaps across subgroups and over time. However, several limitations should be noted. First, the database lacks detailed clinical information such as liver fibrosis stage, HCV genotype, and sustained virologic response data, limiting our ability to fully assess treatment effectiveness and long-term outcomes. Second, the dataset does not capture patient adherence to therapy or specific reasons for treatment refusal, which could provide valuable insights into remaining barriers to care. Third, while we stratified our analyses by key demographic and facility-related factors, unmeasured variables such as patient frailty, socioeconomic status, and healthcare-seeking behavior may have influenced treatment initiation. Fourth, our reliance on the National Health Insurance Laboratory Database carries a risk of ascertainment bias. Because laboratory data submission is voluntary and varies by facility type (eg, lower completeness in freestanding clinics), this systematic variation in laboratory data availability is a key consideration for interpreting the HCV care cascade. Specifically, if facilities with lower reporting completeness also had lower treatment rates, our results may underestimate the true scale of the problem. Finally, our study period ended in 2021, precluding evaluation of the most recent trends.

Notably, open government data indicate that as of March 2025, HCV RNA confirmation among dialysis patients had reached 98% and treatment uptake 96.4% [42], further supporting the success of Taiwan's microelimination strategy. This underscores that the positive trends observed in our study have continued. However, because the dialysis population is dynamic, sustained monitoring, screening, and treatment remain essential to preserve success and prevent re-emergence. In short, achieving high confirmation and treatment rates is a milestone, but maintaining control demands ongoing vigilance.

In conclusion, HCV care among hemodialysis patients in Taiwan has advanced substantially from 2015 through 2021, with high treatment initiation once viremia is confirmed. However, confirmatory RNA testing remains the critical bottleneck, with persistent disparities by region, urbanization, and facility type. As Taiwan approaches its 2025 elimination goal, sustained efforts are needed to maintain these gains and prevent re-emergence. Future research should evaluate the long-term benefits of HCV elimination in this population, including impacts on survival and cardiovascular outcomes, and explore innovative models to close remaining gaps in diagnosis and linkage to care.

## Supplementary Data

Supplementary materials are available at *Open Forum Infectious Diseases* online. Consisting of data provided by the authors to benefit the reader, the posted materials are not copyedited and are the sole responsibility of the authors, so questions or comments should be addressed to the corresponding author.
