# Supplementary material for: The Journey to Hepatitis C Elimination in Taiwan: Insights From the Hemodialysis Population
Source: Open Forum Infect Dis. 2026 Jan 29;13(2):ofag039. doi: 10.1093/ofid/ofag039 (PMC12888814; doi:10.1093/ofid/ofag039)
Supplement: ofag039_Supplementary_Data [file ofag039_supplementary_data.docx]

**Supplemental materials**

**Supplementary Figure S1.** Flowchart of study population selection. Adults aged ≥18 years with chronic hemodialysis for more than three consecutive months between 2015 and 2021 were identified from the Taiwan National Health Insurance Research Database (n=46,644). We excluded individuals who died in 2015 or 2016 (n=4,201) and those who had received HCV treatment prior to initiating hemodialysis (n=585). Among 41,858 eligible patients, anti-HCV antibody data were available for 38,502 (92.0%), who comprised the final study cohort.

**
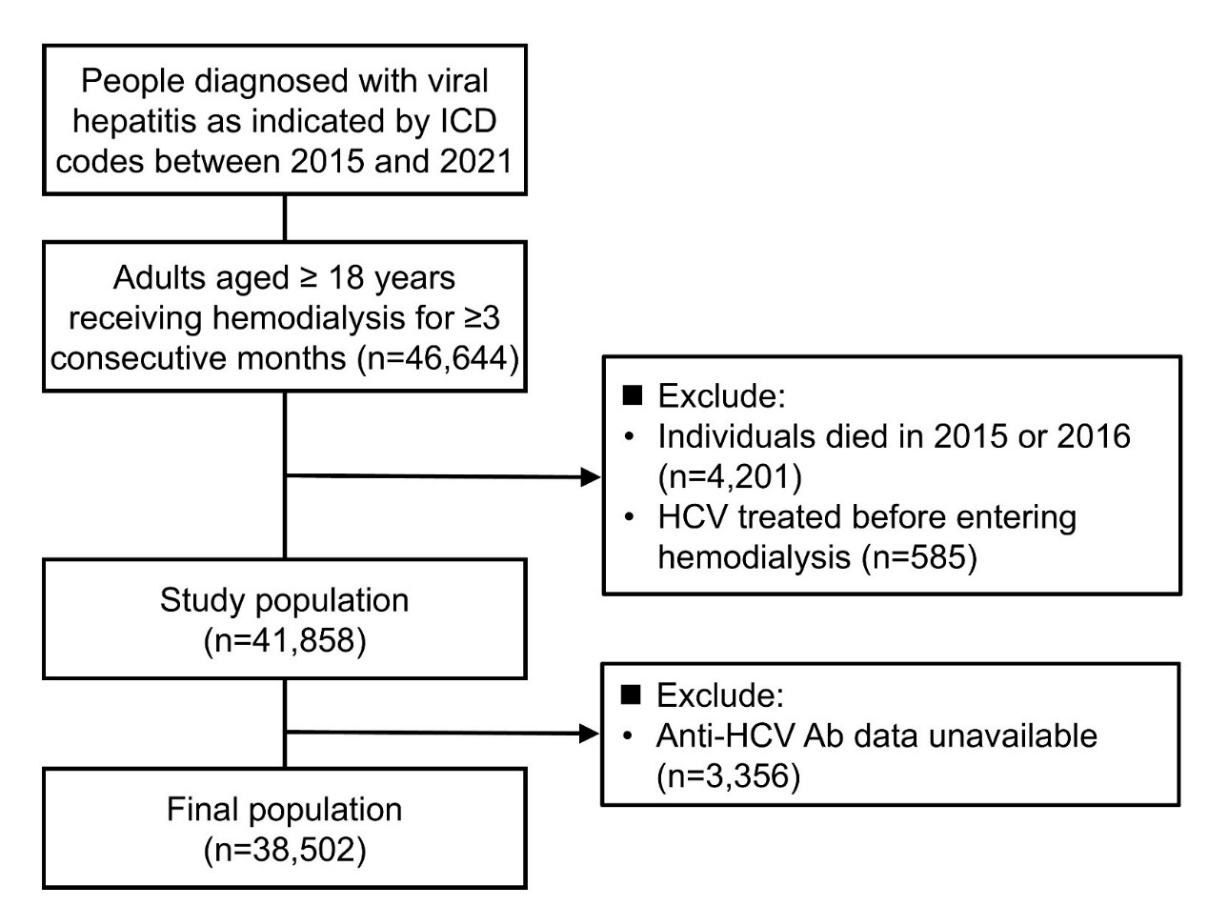
**

**Supplementary Figure S2.** Algorithm for interpreting anti-HCV antibody test results. Text-based results were classified as positive if explicitly stated as "positive" or containing a "+" symbol, and negative if stated as "negative" or containing "-", "borderline," "gray zone," or other non-interpretable text. Numeric results were classified as positive if values met specified cut-offs (≥1 S/CO, ≥1 COI, or ≥2 S/N). When unit or reference range was unavailable, a default cut-off of ≥2 was applied.


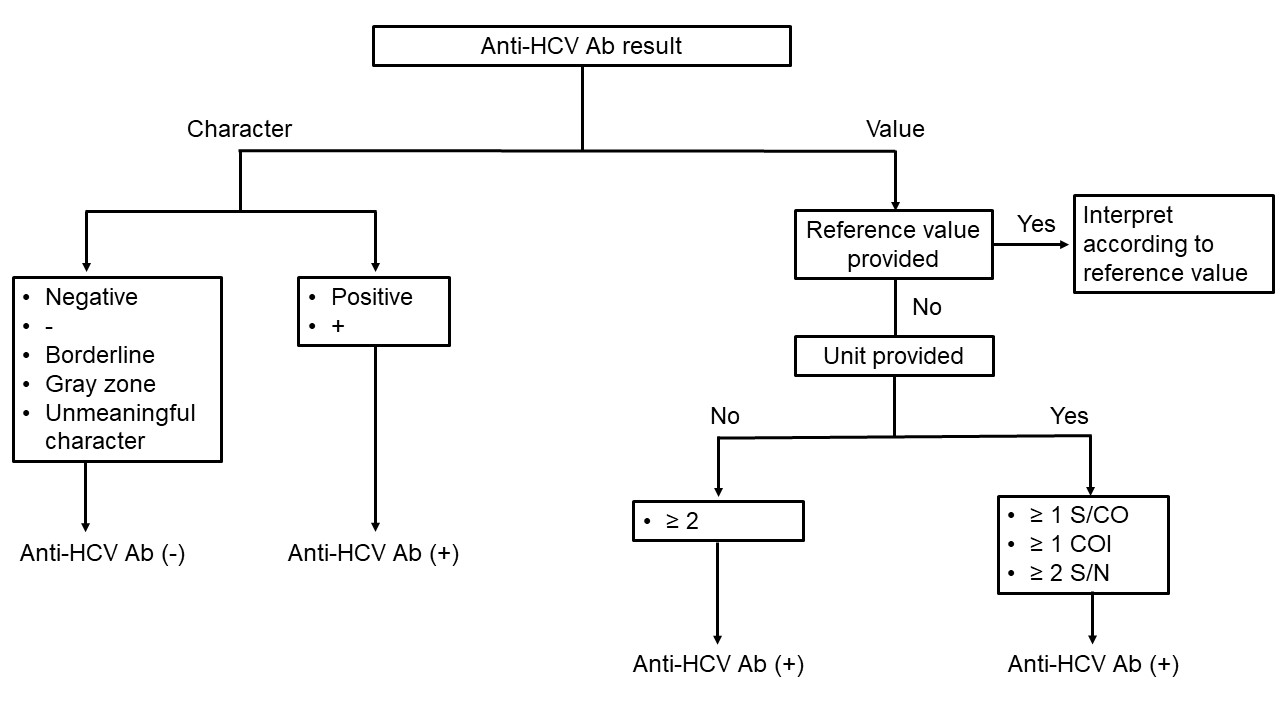


**Supplementary Figure S3.** Algorithm for interpreting HCV RNA test results. Text-based results were classified as positive if explicitly stated as "positive" or containing a "+" symbol, and negative if stated as "negative" or containing "-", "borderline," "gray zone," or other non-interpretable text. Numeric values were classified as positive if ≥10, ≥12, or ≥15 IU/mL when unit and reference range were available. If unavailable, a default cut-off of ≥15 IU/mL was used.

**
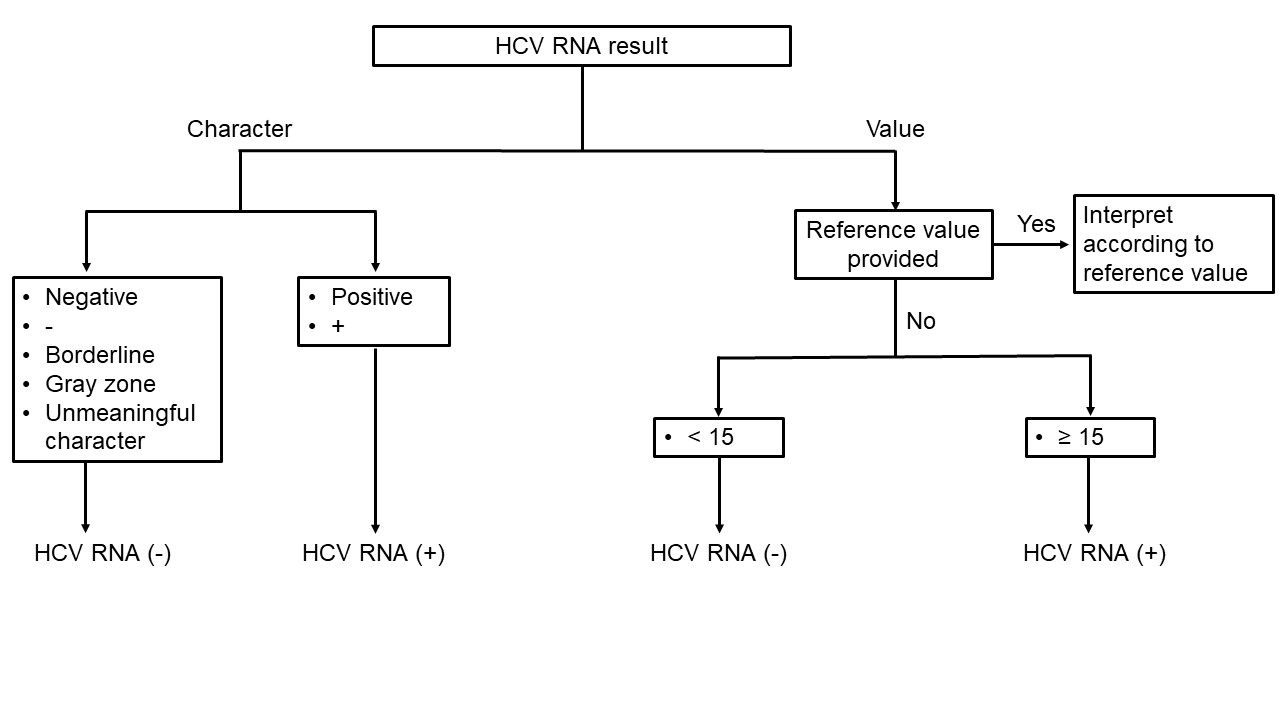
**

**Supplementary Figure S4.** Cumulative rates of confirmatory RNA testing and DAA treatment initiation from 2017 to 2021.

**
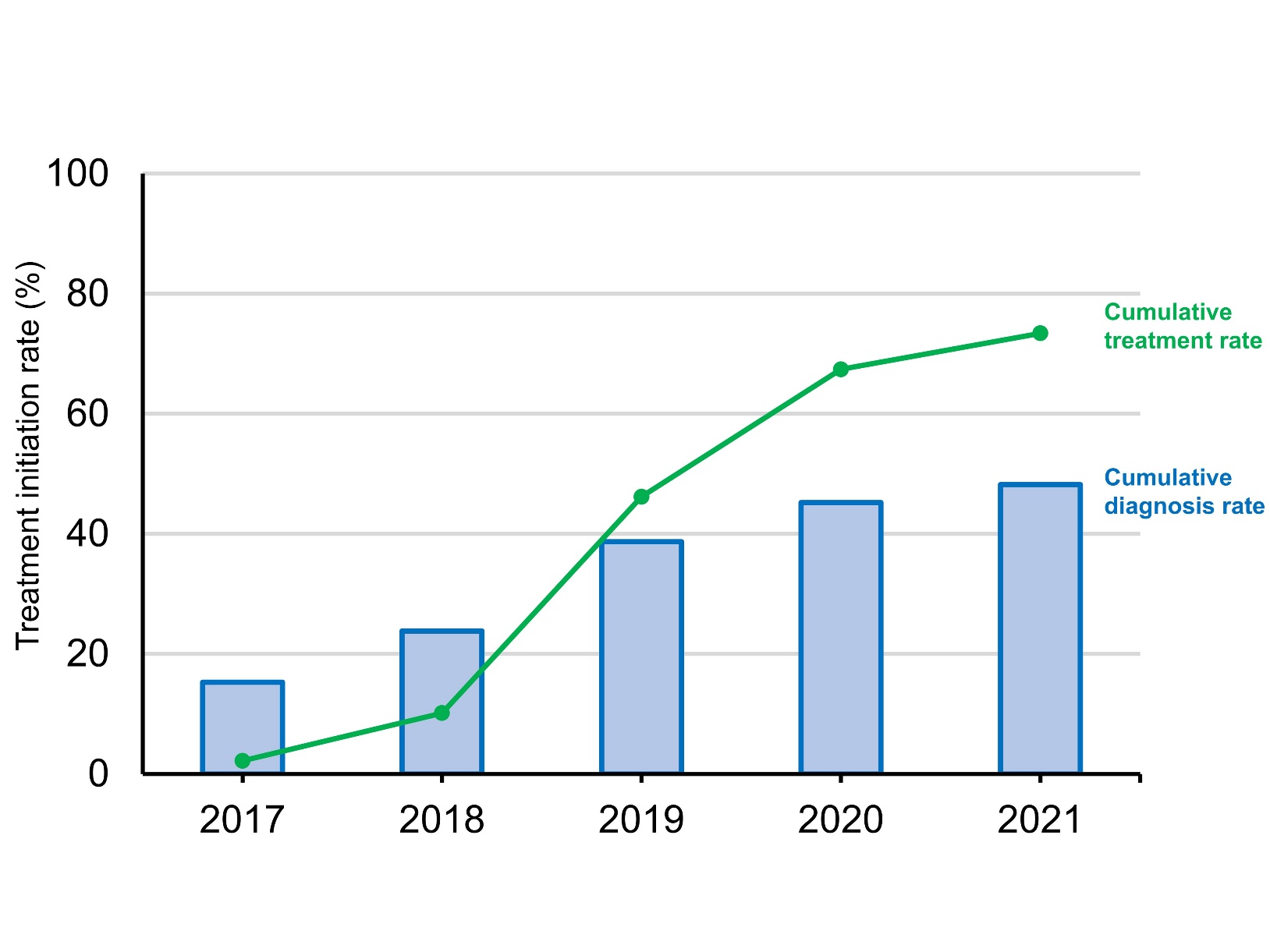
**

**Supplemental Table S1. Data Availability for Anti-HCV and HCV RNA Reporting**

|  | **Total Population  (N)** | **HCV Ab Uploaded  (n)** | **%  (n/N)** | **HCV RNA Checked (n)** | **HCV RNA Uploaded (n)** | **% (Uploaded/Checked)** |
| --- | --- | --- | --- | --- | --- | --- |
| ***Characteristics*** |  |  |  |  |  |  |
| **Total** | 41,858 | 38,502 | 92.0 | 8,315 | 7,112 | 85.5 |
| **Sex** |  |  |  |  |  |  |
| Male | 23,505 | 21,754 | 92.6 | 4,329 | 3,673 | 84.8 |
| Female | 18,353 | 16,748 | 91.3 | 3,986 | 3,439 | 86.3 |
| **Age Group** (years) |  |  |  |  |  |  |
| < 45 | 2,834 | 2,643 | 93.3 | 532 | 463 | 87.0 |
| 45-64 | 18,120 | 16,777 | 92.6 | 4,151 | 3,535 | 85.2 |
| ≥ 65 | 20,904 | 19,082 | 91.3 | 3,632 | 3,114 | 85.7 |
| **Facility Type** |  |  |  |  |  |  |
| Tertiary hospital | 3,558 | 3,521 | 99.0 | 684 | 603 | 88.2 |
| Secondary hospital | 9,375 | 9,059 | 96.6 | 2,039 | 1,739 | 85.3 |
| Primary hospital | 9,361 | 8,893 | 95.0 | 1,853 | 1,639 | 88.5 |
| Freestanding unit | 19,564 | 17,029 | 87.0 | 3,739 | 3,131 | 83.7 |
| **NHI Region ^#^** |  |  |  |  |  |  |
| Taipei | 11,013 | 10,187 | 92.5 | 1,700 | 1,396 | 82.1 |
| Northern | 5,931 | 5,658 | 95.4 | 1,154 | 947 | 82.1 |
| Central | 7,747 | 7,121 | 91.9 | 1,555 | 1,301 | 83.7 |
| Southern | 7,846 | 7,209 | 91.9 | 1,976 | 1,734 | 87.8 |
| Kaopin | 8,090 | 7,199 | 89.0 | 1,614 | 1,445 | 89.5 |
| Eastern | 1,231 | 1,128 | 91.6 | 316 | 289 | 91.5 |

Abbreviations: HCV, hepatitis C virus; Ab, antibody; NHI, National Health Insurance.

**Supplemental Table S2. National Health Insurance (NHI) Order Codes for Direct-Acting Antiviral (DAA) Regimens**

| **NHI Order Code** | **Regimen** | **Treatment Description** |
| --- | --- | --- |
| HCVDAA0001 | Daklinza + Sunvepra | Genotype 1b, 24-week regimen |
| HCVDAA0002 | Viekirax + Exviera | Genotype 1b, without cirrhosis or with compensated cirrhosis, 12-week regimen |
| HCVDAA0003 | Viekirax + Exviera + Ribavirin | Genotype 1a, without cirrhosis, 12-week regimen |
| HCVDAA0004 | Viekirax + Exviera + Ribavirin | Genotype 1a, with compensated cirrhosis, 24-week regimen |
| HCVDAA0005 | Zepatier ± Ribavirin | Genotype 1a, without resistance-associated variants (RAVs), 12-week regimen |
| HCVDAA0006 | Zepatier + Ribavirin | Genotype 1a, with resistance-associated variants (RAVs), 16-week regimen |
| HCVDAA0007 | Zepatier ± Ribavirin | Genotype 1b, 12-week regimen |
| HCVDAA0008 | Zepatier | Genotype 4, 12-week regimen |
| HCVDAA0009 | Zepatier + Ribavirin | Genotype 4, 16-week regimen |
| HCVDAA0010 | Harvoni ± Ribavirin | Genotype 1, 2, 4, 5, or 6, 12-week regimen |
| HCVDAA0011 | Sovaldi + Ribavirin | Genotype 2, 12-week regimen |
| HCVDAA0012 | Maviret | Genotype 1, 2, 3, 4, 5, or 6, 8-week regimen |
| HCVDAA0013 | Maviret | Genotype 1, 2, 3, 4, 5, or 6, 12-week regimen |
| HCVDAA0014 | Maviret | Genotype 1 or 3, 16-week regimen |
| HCVDAA0015 | Epclusa | Genotype 1, 2, 3, 4, 5, or 6, 12-week regimen |
| HCVDAA0016 | Epclusa + Ribavirin | Genotype 1, 2, 3, 4, 5, or 6, 12-week regimen |
| HCVDAA0017 | Vosevi | Genotype 1, 2, 3, 4, 5, or 6, 12-week regimen |

**Supplemental Table S3. Cumulative rate of hepatitis C virus (HCV) RNA testing after a positive HCV antibody result, stratified by city/county and National Health Insurance (NHI) region**

|  | **Positive for HCV Antibody (N)** | **2017**^#^ **n (%)** | **2018 n (%)** | **2019 n (%)** | **2020 n (%)** | **2021 n (%)** |
| --- | --- | --- | --- | --- | --- | --- |
| ***City or County*** |  |  |  |  |  |  |
| **Overall** | 14,755 | 2,257 (15) | 3,507 (24) | 5,710 (39) | 6,675 (45) | 7,112 (48) |
| **Taipei Division** |  |  |  |  |  |  |
| Taipei City | 1,250 | 204 (16) | 308 (25) | 452 (36) | 520 (42) | 569 (46) |
| New Taipei City | 1,755 | 199 (11) | 310 (18) | 517 (29) | 607 (35) | 676 (39) |
| Keelung City | 210 | 14 (7) | 18 (9) | 21 (10) | 31 (15) | 40 (19) |
| Yilan County | 189 | 18 (10) | 28 (15) | 62 (33) | 94 (50) | 102 (54) |
| Kinmen County / Lienchiang County | 52 | 3 (6) | 10 (19) | 14 (27) | 18 (35) | 18 (35) |
| **Northern Division** |  |  |  |  |  |  |
| Taoyuan City | 1,085 | 125 (12) | 194 (18) | 437 (40) | 503 (46) | 527 (49) |
| Hsinchu City | 303 | 32 (11) | 71 (23) | 106 (35) | 123 (41) | 126 (42) |
| Hsinchu County | 193 | 18 (9) | 38 (20) | 71 (37) | 85 (44) | 88 (46) |
| Miaoli County | 407 | 50 (12) | 75 (18) | 164 (40) | 199 (49) | 206 (51) |
| **Central Division** |  |  |  |  |  |  |
| Taichung City | 2,059 | 251 (12) | 370 (18) | 644 (31) | 751 (36) | 791 (38) |
| Changhua County | 710 | 82 (12) | 171 (24) | 332 (47) | 363 (51) | 373 (53) |
| Nantou County | 412 | 36 (9) | 61 (15) | 105 (25) | 131 (32) | 137 (33) |
| **Southern Division** |  |  |  |  |  |  |
| Yunlin County | 747 | 151 (20) | 234 (31) | 351 (47) | 396 (53) | 414 (55) |
| Chiayi City | 492 | 165 (34) | 234 (48) | 294 (60) | 334 (68) | 350 (71) |
| Chiayi County | 329 | 52 (16) | 94 (29) | 153 (47) | 195 (59) | 204 (62) |
| Tainan City | 1,010 | 299 (30) | 413 (41) | 628 (62) | 738 (73) | 766 (76) |
| **Kaoping Division** |  |  |  |  |  |  |
| Kaohsiung City | 2,275 | 373 (16) | 568 (25) | 878 (39) | 1,033 (45) | 1,125 (49) |
| Pingtung County | 525 | 94 (18) | 140 (27) | 246 (47) | 284 (54) | 311 (59) |
| **Eastern Division** |  |  |  |  |  |  |
| Hualien County | 529 | 66 (12) | 117 (22) | 158 (30) | 178 (34) | 191 (36) |
| Taitung County | 223 | 25 (11) | 53 (24) | 77 (35) | 92 (41) | 98 (44) |

Footnotes:

# The data for 2017 include HCV RNA testing performed in 2017 and prior years.

* Denominator for cumulative rates: total number of individuals with a positive HCV antibody test (N = 14,755).

**Supplemental Table S4. Cumulative rate of initiating hepatitis C virus (HCV) treatment after a positive HCV RNA test, stratified by city/county and National Health Insurance (NHI) region**

|  | **Positive for HCV RNA (N)** | **2017 n (%)** | **2018 n (%)** | **2019 n (%)** | **2020 n (%)** | **2021 n (%)** |
| --- | --- | --- | --- | --- | --- | --- |
| ***City or County*** |  |  |  |  |  |  |
| **Overall** ^#^ | 4,783 | 105 (2) | 485 (10) | 2,209 (46) | 3,224 (67) | 3,511 (73) |
| **Taipei Division** |  |  |  |  |  |  |
| Taipei City | 376 | 10 (3) | 30 (8) | 154 (41) | 222 (59) | 250 (66) |
| New Taipei City | 450 | 6 (1) | 36 (8) | 176 (39) | 271 (60) | 311 (69) |
| Keelung City | 33 | 0 (0) | 1 (3) | 9 (27) | 13 (39) | 19 (58) |
| Yilan County | 72 | 1 (1) | 5 (7) | 20 (28) | 58 (81) | 61 (85) |
| Kinmen County / Lienchiang County | 10 | 0 (0) | 0 (0) | 2 (20) | 6 (60) | 6 (60) |
| **Northern Division** |  |  |  |  |  |  |
| Taoyuan City | 381 | 2 (1) | 20 (5) | 197 (52) | 289 (76) | 307 (81) |
| Hsinchu City | 97 | 3 (3) | 13 (13) | 53 (55) | 72 (74) | 75 (77) |
| Hsinchu County | 60 | 1 (2) | 2 (3) | 31 (52) | 37 (62) | 44 (73) |
| Miaoli County | 154 | 1 (1) | 8 (5) | 67 (44) | 113 (73) | 117 (76) |
| **Central Division** |  |  |  |  |  |  |
| Taichung City | 459 | 8 (2) | 51 (11) | 241 (53) | 325 (71) | 350 (76) |
| Changhua County | 240 | 6 (3) | 35 (15) | 166 (69) | 188 (78) | 196 (82) |
| Nantou County | 88 | 2 (2) | 10 (11) | 34 (39) | 57 (65) | 64 (73) |
| **Southern Division** |  |  |  |  |  |  |
| Yunlin County | 276 | 6 (2) | 45 (16) | 143 (52) | 185 (67) | 193 (70) |
| Chiayi City | 243 | 13 (5) | 56 (23) | 143 (59) | 179 (74) | 186 (77) |
| Chiayi County | 135 | 2 (1) | 21 (16) | 64 (47) | 97 (72) | 100 (74) |
| Tainan City | 553 | 11 (2) | 44 (8) | 237 (43) | 388 (70) | 412 (75) |
| **Kaoping Division** |  |  |  |  |  |  |
| Kaohsiung City | 749 | 28 (4) | 79 (11) | 328 (44) | 477 (64) | 539 (72) |
| Pingtung County | 214 | 4 (2) | 14 (7) | 81 (38) | 123 (57) | 140 (65) |
| **Eastern Division** |  |  |  |  |  |  |
| Hualien County | 124 | 1 (1) | 11 (9) | 38 (31) | 79 (64) | 88 (71) |
| Taitung County | 69 | 0 (0) | 4 (6) | 25 (36) | 45 (65) | 53 (77) |

Footnotes:

#: The denominator for overall cumulative treatment initiation rates is the total number of individuals with a positive HCV RNA test (N = 4,783).
